# Self-identification of electronically scanned signatures (ESS) and digitally constructed signatures (DCS)

**DOI:** 10.1080/20961790.2021.1923167

**Published:** 2021-07-05

**Authors:** Zuzanna Kazmierczyk, Ian J. Turner

**Affiliations:** Centre for Excellence in Learning and Teaching, University of Derby, Derby, UK

**Keywords:** Forensic sciences, signature, handwriting, questioned document analysis, electronic signature, digital signature, simulation

## Abstract

The use of electronic signatures as a form of identification is increasingly common, yet they have been shown to lack the dynamic features found in online signatures. In this study, handwritten signatures were scanned to produce electronically scanned signatures (ESS) which were then digitally altered to produce digitally constructed signatures (DCS). The ESS and DCS were presented back to participants to identify which were genuine. Only 1% of participants correctly identified all signatures, with a mean score of 57.6% identifications. The lack of self-recognition of ESS raises questions on their reliability and usefulness as means of personal identification.

## Introduction

Signatures are widely used tools for personal identification, the confirmation of authorship and the authentication and verification of documents [1]. Signatures are highly individualized habitual writing acts that require minimum concentration to produce [1]. Traditionally the work of a forensic document examiner (FDE) focused exclusively on manuscript-based, handwritten signatures (HS). The increasing use of electronic signatures has presented challenges to FDEs in their approaches to examination, due to limited standardized methodologies, research in the area and difference between them and inked signatures [2,3].

Electronic signature is a broad term that includes: digital-based algorithm-derived signatures, biodynamic signatures produced on an electronic device which is a representation of an HS and electronically scanned versions of handwritten signatures (ESS) [2,4]. The Electronic Signatures in Global and National Commerce Act (2000) in UK states that an electronic form of a signature (or contract, or other form) may not be “denied legal effect, validity, or enforceability because it is in electronic form”.This law makes electronic signature as enforceable and as binding as a traditional written signature.

Verification of electronic signatures can take place offline and online. Offline signature verification uses images of the signature that are processed on either a computer programme or by an FDE [5]. In offline verification, it is reported that many of the dynamic features of signature construction, normally analysed in a written signature, are lost [3,5–7]. Online veri­fication uses data taken directly through the stylus or digital device and generates dynamic values based on kinetic (or biodynamic) parameters. The use of temporal data such as pen speed can provide information that can only be estimated in manuscript signatures. There are few scientific studies that utilize these biodynamic parameters for forensic ana­lysis [8–11].

Simulated signatures are those which attempt to replicate a genuine signature and all its dynamic features. They are generally either “freeform” copied from a genuine specimen or “traced” using a lightbox, sharp implement or pencil to create an impression of the genuine signature to guide the simulation [4,12,13]. It has been demonstrated using MovAlyzeR® software that stroke duration, velocity, and pen pressure can be used to discriminate between genuine and simulated signatures, irrespective of the writing style of the author which was not the case for smoothness (jerk) or size [14]. It has likewise been shown that dynamic features such as signature size, trajectory and speed were the most reliable features for identifying the difference between simulated and genuine electronic signatures, and that dynamic information can be used to connect separate simulation cases [13].

There is limited study on ESS and the simulation of such signatures (either prior to, or post the scanning process). It has been identified that a range of features used in the analysis of HS are lost in the scanning process [3] and that the choice of writing implement can affect the amount of information lost. In a study of 16 participants the difference between signatures produced by writing with a ballpoint pen, a digital tablet pen and a computer mouse was examined. The authors found there were significant differences between the temporal and spatial dimensions; in both the online and offline signatures, intra-writer and inter-writer, and between the digital pen and mouse [15].

The ability of FDEs to distinguish simulated and genuine signatures has been compared to lay groups in blind experiments, and all the studies provide evidence that the FDEs have a clear superior ability in identification [16–19]. These studies all adopt specimen signatures as the subject of their experiments, there are no reported studies where authors were asked to self-identify (or other groups evaluate the authenticity of) simulations of their own signature.

Identifying one’s own signature could become more important as ESS are increasingly used as a form of identification and a record of a “genuine” signature, for example, the Identity Card (USA) and Driving License (UK). If these are the reference points for examination by lay people or even FDEs then they pose a potential risk. A simulated signature produced from an ESS, either a digitally constructed signature (DCS) or a handwritten one, with the loss of dynamic features may increase the use of these forms of identification for fraudulent purposes. This paper aims to understand how efficient the lay public are at identifying their own ESS from a pool of genuine and DCS variants.

## Materials and methods

The study was conducted under the ethical approval of the University of Derby.

**Figure 1.**
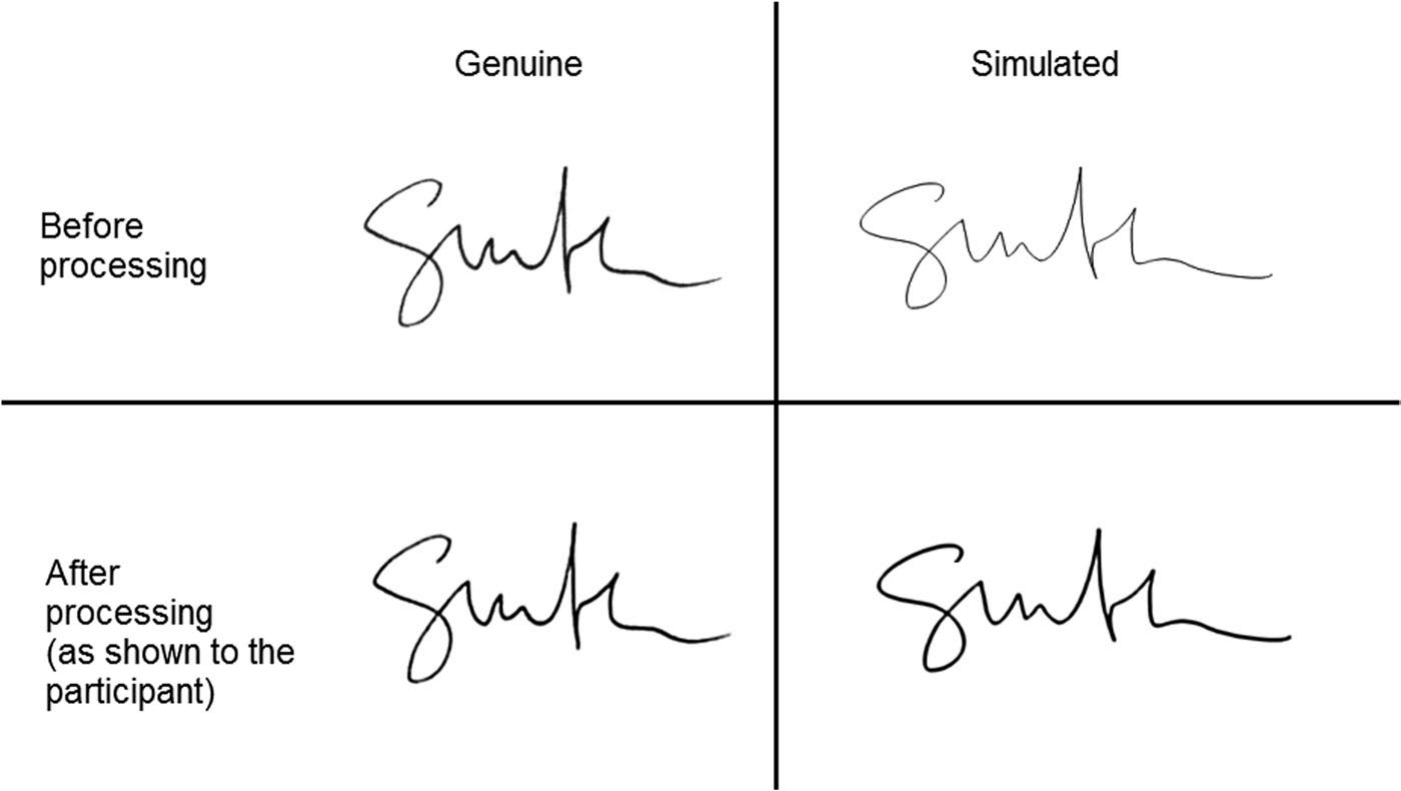
A handwritten signature (HS) was electronically scanned (top left) and simulated by tracing onto a Wacom CTL using a digital stylus (top right). The signature were resized and pixel removed (digitally constructed signature, DCS) only to match the HS (bottom left and right).

One hundred participants, with no experience in signature examination, provided 10 signatures on a sheet of plain white paper (90 g/m^2^) using a black ballpoint pen (Parker, Newhaven, UK). The participants were aged 18–55 and had variable levels of educational background. The HS were then: (1) scanned at 300 dpi to produce ESS and placed on a Wacom CTL-480 Graphics Tablet (Kazo, Japan) and (2) copied by the experimenter using the stylus to produce DCS. The DCS were size-adjusted and pixilation was removed using PhotoFiltre 7 (Houilles, France) and Microsoft Office Picture Manager (https://support.microsoft.com/en-us/topic/about-picture-manager-f767aca9-e818-4dfc-b71a-f2184d6bbde9). This process took on average 6 min per signature and was undertaken with the purpose of making the signatures a replica of the HS (Figure 1).

A random set of 10 signatures from the pool of available ESS and DCS, were presented to each participant at least 1 week after the original collection. Participants viewed, with the naked eye, the signatures one at a time and were asked to identify if they were genuine or a simulation. Post activity, a point was given for a correct identification with a maximum of 10 (i.e. 100%). Participants were informed the sample contained between 0–10 genuine signatures before the identification and shown their score once they had completed the task.

Sixteen participants chosen at random were asked to repeat the study, but with a copy of their HS available for comparison. The same scoring system as in the main study was used, in addition participant’s perception of the task was recorded. Additionally, signatures of each participant were classified as either difficult or easy to forge. The classification was based on the opinion of the author performing the simulations.

## Results

Only one out of the 100 participants was able to correctly identify 100% of their signatures (Table 1). The mean score for all participants was 57.6%. Forty-one participants had a result below 60%, 30 above and 29 achieved exactly 60%. The ratio of genuine/simulated signatures in the sample did not have a bearing on the ability of participants to correctly identify their own signatures.

**Table 1.** The number of participants and the percentage of sample signatures they correctly attributed as genuine/simulation.

Score (%)	Number of participants
20	2
30	5
40	17
50	17
60	29
70	16
80	9
90	4
100	1

The total number of questioned signatures in the project was 1 000 (100 participants, 10 signatures each). Among them there were 550 genuine signatures, of which 309 (56.2%) were correctly recognized to be genuine. Out of 450 simulated signatures, 267 (59.3%) were correctly identified as simulations. Only 25 participants correctly identified all forged versions of their signatures (Table 2).

**Table 2.** The ratio of genuine and simulated signatures provided to participants and their corresponding accuracy in identification.

Signature set		Number of participants	Average result (%)
Genuine	Simulated		
0	10	10	60.0
1	9	10	59.0
2	8	6	60.0
3	7	4	57.5
4	6	3	53.3
5	5	12	52.5
6	4	6	68.3
7	3	15	56.7
8	2	13	57.7
9	1	11	56.4
10	0	10	56.0

Signatures of 61 participants were classified as easy to forge and the remaining 39 were classified as difficult to forge. The mean results of participants were 56.6% and 59.2% for the easy and difficult to forge groups, respectively.

Subsequently, 16 participants were questioned twice, with their HS provided for comparison (Table 3). Half (50%) of the participants’ ability to identify signatures as genuine or simulated improved (three participants correctly identified all). Whereas 50% showed no improvement or decrease in correct identifications.

**Table 3.** Participants’ ability to identify signatures as genuine or simulated without (1st attempt) and with (2nd attempt) a hard copy specimen.

Participant code	Score (%)		
	1st attempt	2nd attempt	Difference
1	60	40	−20
2	60	50	−10
3	80	70	−10
4	60	70	10
5	60	30	−30
6	60	60	0
7	50	40	−10
8	40	70	30
9	40	100	60
10	90	90	0
11	100	90	−10
12	50	70	20
13	40	60	20
14	40	70	30
15	70	100	30
16	60	100	40

## Discussion

The participants correct identified on average 57.6% of the signatures’ origins (genuine or simulated) from a sample they examined. The ratio of genuine to simulated signatures they were provided with made little difference to the overall recognition. This value is similar to that reported by Found et al. [16] where the lay group correctly identified 57.1% of signatures, although in that case, participants could declare an inconclusive opinion after examination. The explanation for participants not being able to identify their own signature could be unfamiliarity with the subject matter. However, it was the experimenter’s view the findings were due to the strength of the simulations.

Providing participants with genuine hard copy of their own signature did improve identification in half of participants. It was noted that some participants made (incorrect) judgements because they thought they could see an exact tracing of their signature on the screen when compared to the one on the specimen sheet. It could be that complexity of the signature, and consequently ability of the simulator to imitate was a factor. The mis-identification illustrates the ease with which an ESS can be used to produce a DCS and fool the originator of the signature. Given the legal validity of electronic signatures it may be of concern to individuals that they are unable to recognize their own. FDEs have demonstrated a superior ability to lay groups when distinguishing between genuine and simu­lated signatures [16–19]. However, if self-identification is not consistently possible it raises concerns about the lay people required to make authentication judgements about these documents in the real world, e.g. governmental and banking employees.

It has been demonstrated that the dynamic features of electronic signatures are lost in offline veri­fication [3,6]. Online verification with its range of dynamic values offers rich information for analysis by FDEs. However, ESS and DCS illustrate that a signature presented in a digital format cannot be recognized consistently by the originator raising a potential vulnerability to this form of identification. It raises a potential complication to FDEs when an author cannot identify a genuine “specimen” sample for comparison purposes.

## Authors’ contributions

Zuzanna Kazmierczyk collected the data. Zuzanna Kazmierczyk and Ian J. Turner analysed the data and prepared the manuscript.
